# How fixed fees and patient choice can support eye care for the poorest

**Published:** 2013

**Authors:** Raheem Rahmathullah

**Affiliations:** Director of Sustainability Initiatives: International Eye Foundation, Kensington, MD, USA. **raheem@iefusa.org**

**Figure:**
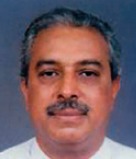
Raheem Rahmathullah

Clinica Oftalmologica Divino Nino Jesus (DNJ) in Peru exemplifies how one small eye clinic can become sustainable, grow, and influence national eye care strategies.

DNJ was established in 1996 and initially provided general health care. From 2006 onwards, CBM Latin America helped DNJ to focus on eye care and later sponsored a DNJ leadership team to attend an International Eye Foundation (IEF) sustainability workshop in Paraguay. DNJ then started a two-year change process toward sustainability with technical assistance from IEF and Guatemala's Visualiza.

## Leadership commitment

IEF worked with the hospital management to develop a business plan which included:

- a focus on patient needs and expectations
- the creation of product and service choices
- good management practices
- standardisation of protocols.

The budget focused on:

- procurement
- cost reduction
- pricing structures
- patient willingness to pay.

Overall, the business plan relied on a detailed analysis of current capacity and an understanding of future demand for services, including what people might be willing to pay.

Initially, there was some resistance from the leadership and staff to charging fees for services, as most patients had been treated free of charge (except for a small number of private patients).

**Figure:**
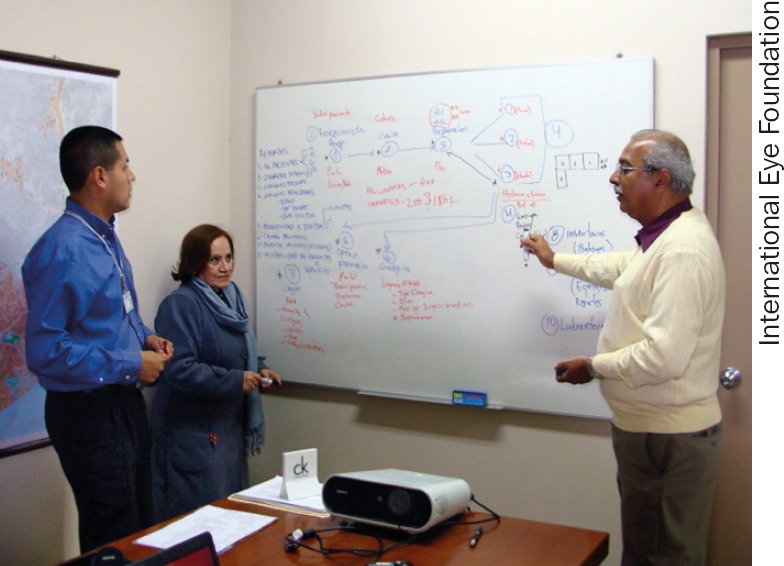
Planning sustainable services at Clinica Oftalmologica Divino Nino Jesus.

At DNJ, the following product and service choices have been introduced:

- A standard intraocular lens (IOL) vs. a more expensive foldable IOL.
- ‘First come, first served’ or ‘fast track’ when attending outpatient examinations (see opposite page for more detail).

## Who is subsidised?

To determine what percentage of patients should be subsidised – and what percentage can be treated free – DNJ used a formula based on estimated population income.

**Rich or very rich patients** (5% of the population) can choose to pay for foldable IOLs and fast track, which have a higher fee. This means that the hospital makes a bigger profit from these patients and the profit can then be used to subsidise the poor and very poor patients.

**Middle-income patients** (55% of the population) cover the full cost of their care: the basic cost of consumables plus the costs of overheads such as staff time, electricity, and other related costs.

**Poor patients** (30% of the population), pay a reduced fee which covers only the cost of consumables; DNJ subsidises them by paying the overhead costs.

**Very poor patients** (10% of the population) receive services free – they are fully subsidised. DNJ pays all the costs associated with their care, whether through its own revenue or through a government subsidy or charitable donation. Patients referred by the outreach programme are also treated without charge. This has built the reputation of the ‘new DNJ’.

**Overcoming resistance to change**

In our experience, gaining the trust of people in key leadership positions is possible but can be slow; it involves carefully discussing any sensitive issues. Relationships have to be carefully nurtured.

The key to overcoming resistance is to change the way these leaders think. Help them to see the benefits of the proposed change by linking the result of the changes to the vision, mission, goals and financial returns of the organisation.

The leaders must develop confidence in the change process. Help them to understand what is being done and point out any areas of progress as they occur. It is these incremental successes that increase confidence in the consultant and the process itself.

If there are problems within the organisation itself, we find that it is best to discuss these at two levels:

1. At the senior management level.
2. At the staff level, with one senior management person present. A facilitator can then elicit comments from all staff in an open and frank discussion.

**Getting the price right**

**Fixed prices vs. negotiated prices**

Many clinics negotiate prices with patients; however, fixed prices are more effective. A clear list (or menu) of services, at affordable prices, will increase patient volume and thus profit. The list can contain different types of services at different prices.

Fixed prices are more convenient and enable the patient to arrive with the correct amount of money. Patients should be able to pay for the entire service (pre-operative services, surgery and post-operative follow-up) as one package, rather than facing repeated charges for return visits or being charged an unexpected fee at every station.

Another advantage of fixed pricing is that the person providing advice to the patient (sometimes called a ‘patient counsellor’) will not be wasting time negotiating prices. Rather, the counsellor can describe the services offered and help the patient decide which type of service he or she wishes and is willing to pay for.

To arrive at the correct fixed price, look at the average of the negotiated prices for each service, and take that average to be the new fixed price. Each service will be affordable to most people.

**Pricing based on product and service choices**

The clinic must guarantee that all patients receive eye care in a convenient way and in a safe environment. These are the basics of quality care. However, many patients choose to pay for amenities beyond the basics. At Clinica Oftalmologica Divino Nino Jesus, patients can choose to pay more to go to the front of the queue (the ‘fast track’ option) or to have a foldable IOL used in cataract surgery. These options are perceived as being of greater value and patients will pay higher prices.

## Providing a better service

- Surgical patients are asked to come at appointed times, in ‘batches’, to reduce waiting time and apprehension before surgery. The times are determined by the number of operations done per hour.
- To further reduce apprehension, the counsellor who saw the patient in the outpatient department greets the patient at the operating room, waits, and takes them back to their family member(s) after surgery. The counsellor gives post-operative care advice before the patient goes home. We find that good customer service reduces fear and increases surgical acceptance rates and patient satisfaction.
- Visualiza helped to refine the manual small-incision cataract surgical techniques used by DNJ's surgeons. This improved the quality of surgery and increased the number of cataract operations per hour from two to seven.
- The operating room floor plan was reorganised to increase patient flow from ansaesthesia to the surgical table. This resulted in the surgical roster being completed by 11:00 a.m. instead of 1:00 pm, allowing more operations to be performed in a day.
- Outpatient examinations are scheduled every day. On arrival, patients may choose the ‘fast track’ option at a higher price to be seen quicker. This option reduces patients in the waiting area, allowing more patients in.

### Computerised management information system

To monitor all aspects of DNJ's service delivery and management, a Spanish-language computerised management information system, which was developed by Visualiza, was installed.

## Increasing the number of patients who pay

In 2008, DNJ provided cataract surgery at only two price levels. One was free of charge and the other was an expensive private patient fee. In Figure 2, the brown section represents a very few patients who would essentially have been treated free of charge, but had subsidies from donors or other outside support which contributed toward the cost of surgery. By fixing fees according to patient income, many of the patients who would normally fit into the free category were now able to cover all or some of the costs of their care. Offering patients a choice of products and services, based on perceived value, has also boosted income.

Figures 1 and 2 reflect:

- an increase in cataract surgery
- an increase in the number of patients covering the cost of surgery
- a decrease in the number of patients who were subsidised at 100% (receiving their operation free).

DNJ is now a national leader in eye health in Peru. It collaborated with the Clinton Foundation's cataract surgery initiative, VISION 2020 Latin America, and provides technical assistance to four CBM/LA supported hospitals. DNJ helps develop eye care delivery standards for Peru, coordinates workshops and courses, and is a technical resource for ministry of health ophthalmic training programmes. It has come a long way in the last 7 years.

**Figure:**
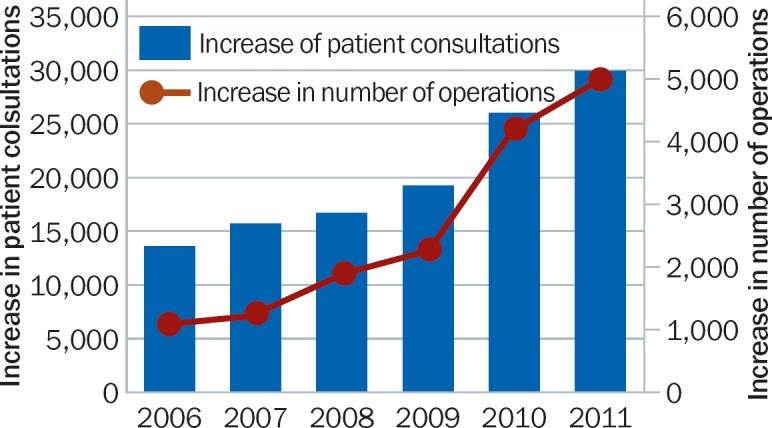
Figure 1. Increase in patient consultations and operations, 2006–2011

**Figure:**
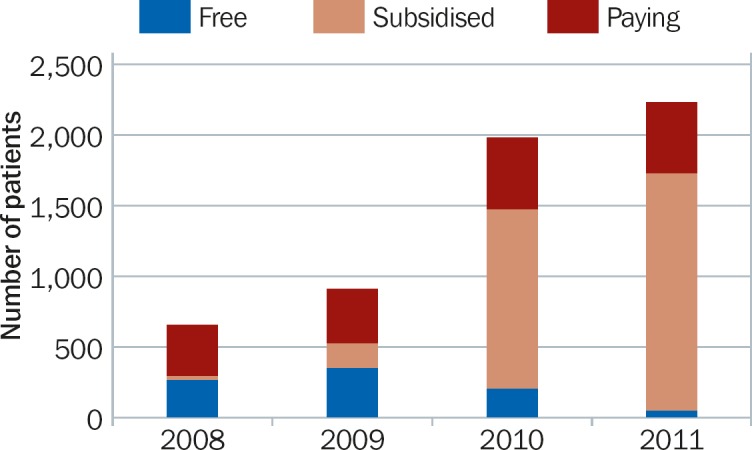
Figure 2. Increase in paying patients, 2008–2011

